# Suicide Protective Factors Among Trans Adults

**DOI:** 10.1007/s10508-013-0099-8

**Published:** 2013-04-24

**Authors:** Chérie Moody, Nathan Grant Smith

**Affiliations:** Department of Educational and Counselling Psychology, McGill University, 3700 McTavish St., Montreal, QC H3A 1Y2 Canada

**Keywords:** Trans, Transgender, Transsexual, Suicide, Protective factors

## Abstract

A recent study indicated a suicide attempt rate of 41 % among trans (e.g., trans, transgender, transexual/transsexual, genderqueer, two-spirit) individuals. Although this rate is alarming, there is a dearth of literature regarding suicide prevention for trans individuals. A vital step in developing suicide prevention models is the identification of protective factors. It was hypothesized that social support from friends, social support from family, optimism, reasons for living, and suicide resilience, which are known to protect cis (non-trans) individuals, also protect trans individuals. A sample of self-identified trans Canadian adults (*N* = 133) was recruited from LGBT and trans LISTSERVs. Data were collected online using a secure survey platform. A three block hierarchical multiple regression model was used to predict suicidal behavior from protective factors. Social support from friends, social support from family, and optimism significantly and negatively predicted 33 % of variance in participants’ suicidal behavior after controlling for age. Reasons for living and suicide resilience accounted for an additional 19 % of the variance in participants’ suicidal behavior after controlling for age, social support from friends, social support from family, and optimism. Of the factors mentioned above, perceived social support from family, one of three suicide resilience factors (emotional stability), and one of six reasons for living (child-related concerns) significantly and negatively predicted participants’ suicidal behavior. Overall, these findings can be used to inform the practices of mental health workers, medical doctors, and suicide prevention workers working with trans clients.

## Introduction

Suicide is a serious, preventable, global health problem (World Health Organization, 2011). The WHO estimates that almost 1 million people die by suicide globally per year. Furthermore, the WHO estimates that suicide attempts occur approximately 20 times more frequently than completed suicides. Current lifetime suicide attempt rates in adults are estimated to be between 1.9 and 8.7 % in the U.S. and between 0.4 and 5.1 % worldwide (Nock et al., 2008).

### Suicide Attempts in Lesbian, Gay, and Bisexual (LGB) Communities

It has been well-established that being a sexual or gender minority puts one at greater risk for suicidal thoughts and behaviors (Haas et al., 2011; King et al., 2008; McDaniel, Purcell, & D’Augelli, 2001). Significant relationships have been documented between same-sex behavior/sexual orientation and suicide attempts, both in LGB youth (see Marshal et al., 2011) and LGB adults (see King et al., 2008). One literature review identified the suicide attempt rates in LGB individuals to be between 20 and 53 % (McDaniel et al., 2001).

It is important to note that the majority of the studies that have investigated suicidal ideation and attempts in LGB individuals have made references to this phenomenon being due, at least in part, to societal anti-LGB opinions, internalized homophobia, stigma, rejection, and discrimination, with some studies testing this hypothesis directly. Relatedly, Meyer (1995, 2003) noted that LGB mental health problems (including suicidal ideation and attempts) are related to minority stress, the chronic stress of living in homophobic social environments. Moreover, the recent National Strategy for Suicide Prevention (2012) released by the U.S. Surgeon General and the National Action Alliance for Suicide Prevention explicitly outlines the link between minority stress and suicidal behaviors for LGB and T (trans^1^) individuals.

### Suicide Attempt Prevalence Rates in Trans Populations

A number of large- and small-scale needs assessments have been conducted in the U.S. that provide information regarding suicide attempts in trans individuals. Results from two needs assessments conducted in Philadelphia showed that 30.1 % of the 182 participants had attempted suicide at least once (Kenagy, 2005). A needs assessment conducted in Chicago found that 27 % of the 108 participants had attempted suicide (Kenagy & Bostwick, 2005). Lastly, a needs assessment conducted with trans people of color in Washington, DC showed that 16 % of participants had attempted suicide at least once (Xavier, Bobbin, Singer, & Budd, 2005).

Results from studies using convenience samples show similar suicide attempt rates. In a sample of 55 trans youth, 26 % of participants had attempted suicide at least once (Grossman & D’Augelli, 2007). Among trans adults, studies have found suicide attempt rates of 23.3 % (Mathy, 2002), 28–31.2 % (Nuttbrock et al., 2010), and 32 % (Clements-Nolle, Marx, & Katz, 2006). In Canada, among a sample of 433 trans individuals living in Ontario, the lifetime suicide attempt rate was 43 % (Scanlon, Travers, Coleman, Bauer, & Boyce, 2010). In a sample of individuals from Minnesota, 47 % of trans participants reported having considered or attempted suicide in the last three years, which was a significantly higher rate when compared to the other sexual minority participants (Bockting, Huang, Ding, Robinson, & Rosser, 2005). The National Transgender Discrimination Survey, a recent nation-wide U.S.-based survey of 6,456 self-identified transgender/gender non-conforming individuals, found that 41 % of participants reported attempting suicide at least once (Grant et al., 2011). For a summary of the literature regarding suicide attempt rates in trans populations, please see Ramsay (n.d.).

### Suicide Risk Factors Among Trans Individuals

Many of the studies mentioned above have examined suicide risk factors among trans adults. For instance, Mathy (2002) found a non-significant difference in suicide attempt rates between trans individuals and lesbians, which led Mathy to draw a parallel between the sexism and heterosexism experienced by both these groups. Mathy concluded that these forms of oppression may be elements that place both trans individuals and lesbians at risk for suicidal ideation and behavior. Mathy also found significant differences between trans participants who had attempted suicide and those who had not, with attempters reporting higher rates of prior and current therapy, prior and current psychiatric medication, and past problems with alcohol and drug use.

Clement-Nolle et al. (2006) found that younger age, depression, past alcohol or drug treatment, forced sex or rape, gender discrimination (being discriminated against due to one’s gender identity/presentation), and physical gender victimization (being beaten or physically abused due to one’s gender identity/presentation) were each independent predictors of attempted suicide. Nuttbrock et al. (2010) examined the relationships between physical gender-related abuse, psychological gender-related abuse, and suicidal ideation and behavior across five life stages of trans participants. Significant associations were found between both types of abuse and suicidal ideation and behavior during early and late adolescence for the 19–39 year-old participants, as well as during all life stages for the 39–59 year-old participants, with the exception of psychological gender-related abuse in the early-young adult life stage.

Scanlon et al. (2010) found that, of the participants who reported experiencing suicidal ideation in the past year, 47 % had experienced physical or sexual assault at some point in their lives due to being trans. Of the participants who reported attempting suicide in the past year, 29 % had experienced physical or sexual assault at some point due to being trans.

### Protective Factors

To date, there exist no published studies examining suicide protective factors among trans individuals. In addition to identifying risk factors, it is important to identify protective factors for at-risk individuals (Eisenberg & Resnick, 2006; Wang, Lightsey, Pietruszka, Uruk, & Wells, 2007). Suicide protective factors are generally those factors that lower the risk of suicide (White, 1998) or that help a person defend against suicidal behaviors (Rutter, Freedenthal, & Osman, 2008). It is important to note that protective factors are not simply the absence of risk factors (Cha & Nock, 2008) nor are they simply the opposite of risk factors (Gutierrez & Osman, 2008). Empirically identifying suicide protective factors, when combined with risk factors, can therefore lead to the development and improvement of suicide prevention models and interventions (Gutierrez & Osman, 2008).

The purpose of the present study was to examine suicide protective factors in trans adults by identifying factors that are negatively associated with suicidal behavior in this population. Although risk factors were not combined with protective factors, it is our hope that this study is a first step in identifying possible protective factors that can then be combined with risk factors in future research and suicide prevention models.

A vast number of protective factors for cis individuals (cisgender and cissexual individuals, which refer to non-trans individuals) have been identified in existing literature. A complete review of all of the protective factors that have been identified is beyond the scope of this article; however, certain well-tested or promising protective factors will be reviewed, including their theoretical or empirical associations to LGB suicidal ideation and attempts.

#### Reasons for Living

Reasons for living, as operationalized by Linehan, Goodstein, Nielsen, and Chiles (1983), are “life-oriented beliefs and expectations that might mitigate against committing suicide” (p. 277). Linehan et al. postulated that certain beliefs, namely reasons for not committing suicide, were an important factor that differentiated suicidal people from non-suicidal people. Linehan et al. have been credited with being the first to operationalize and measure suicide protective factors through their development of the Reasons for Living Inventory (Rutter, 2008).

The examination of reasons for living among LGB populations has been carried out in a small number of studies (e.g., Hirsch & Ellis, 1998; McBee-Strayer & Rogers, 2002). These studies have found that, compared to heterosexual participants, sexual minority participants reported significantly fewer reasons for living related to most or all categories. Hirsch and Ellis contextualized their findings in the stigma and minority status experienced by gay and lesbian individuals. They postulated that stigma and minority status may lead to gay men and lesbians having a more difficult time coping with their environments when compared to heterosexuals, which may lead to fewer reasons for living. On the other hand, it has recently been postulated that perhaps the lower endorsements of reasons for living among sexual minorities is related to the measurement of reasons for living. McBee-Strayer and Rogers conducted an exploratory factor analysis on the Reason for Living (RFL) Scale in order to test its construct validity when used with sexual minorities. Their results did not support the scale’s reported structure. A recent qualitative exploration (Garrett, Waehler, & Rogers, 2012) of 19 LGBT individuals’ perceptions of the RFL indicated that some of the scale’s items may not capture sexual minorities’ reasons for living or may be ambiguous for LGBT individuals. Despite these challenges, alternative scales for measuring reasons for living in LGBT samples are lacking. In addition, it is unknown how reasons for living are related to suicidal behavior in trans individuals. As such, though there are measurement limitations, the current study sought to examine reasons for living and their association with suicidal behavior in a sample of trans individuals.

#### Suicide Resilience

Suicide resilience is a relatively new protective construct that has emerged in the suicide literature. It is defined as “the *perceived* ability, resources, or competence to regulate suicide-related thoughts, feelings, and attitudes” (Osman et al., 2004, p. 1351).

Suicide resilience has yet to be investigated in LGB populations. It has, however, been integrated into a conceptual model regarding LGB youth suicide. Rutter (2008) developed the cumulative factor model, in which both risk and protective factors were taken into account when examining suicidal ideation and attempts in LGB youth. Rutter proposed that the examination of the intersection of certain protective factors (social support, suicide resilience, and optimism) and known risk factors for LGB youth suicide (mental health problems, substance abuse, and sexual orientation victimization) may lead to improved suicide assessments and interventions for LGB youth. The cumulative factor model was not tested in the current study; however, it is mentioned here due to the fact that it clearly illustrates the importance of identifying protective factors, as well as pointing to a number of protective factors that may be beneficial to investigate.

#### Social Support

Social support is considered to be an important suicide protective factor (Goldsmith, Pellmar, Kleinman, & Bunney, 2002; Gutierrez & Osman, 2008; Nock et al., 2008). Many studies have examined the relationship between social support and suicidal ideation and/or attempts, with results indicating that social support is a negative predictor of suicidal ideation and attempts. For instance, social support negatively predicted suicidal ideation in a sample of African American female college students (Marion & Range, 2003) and it negatively predicted suicidal ideation above and beyond hopelessness and depression in a sample of Norwegian undergraduate students (Chioqueta & Stiles, 2007). Social support has been shown to be a suicide protective factor for both LGB youth (Eisenberg & Resnick, 2006; Fenaughty & Harré, 2003) and older adults (D’Augelli, Grossman, Hershberger, & O’ Connell, 2001).

#### Optimism

Dispositional optimism is a stable trait that “reflects the extent to which people hold generalized favorable expectancies for their future” (Carver, Scheier, & Segerstrom, 2010, p. 879). A small number of studies have examined the relationship between dispositional optimism and suicidal ideation and/or attempts. Results have been mixed; some studies have shown support for optimism as a protective factor (Hirsch, Conner, & Duberstein, 2007; Rasmussen & Wingate, 2011) while others have not (Hirsch & Conner, 2006). Optimism has yet to be investigated in LGB populations, but was integrated into Rutter’s (2008) conceptual cumulative factor model as a protective factor for LGB youth.

### Purpose and Hypothesis

There is an absence of empirical data regarding suicide protective factors in trans populations. Given the high suicide attempt rates that have been documented in trans communities, the investigation of protective factors appears to be overdue.

It was hypothesized that optimism, perceived social support from friends, and perceived social support from family will negatively predict suicidal behavior in trans adults. Furthermore, it is hypothesized that reasons for living and suicide resilience will also negatively predict suicidal behavior in trans adults, above and beyond optimism, perceived social support from friends, and perceived social support from family.

## Method

### Participants

The data used in the present analyses were collected between September 2010 and February 2011. It was not possible to determine how many participants began the survey and subsequently withdrew their consent due to the fact that participants had the option of clearing their responses before exiting the survey platform. A total of 134 participants completed and submitted the questionnaires. One participant experienced computer difficulties (this was explained by the participant in the comments section of the questionnaire) and was subsequently only able to complete the demographic questions and one other scale. This participant’s responses were not included in the final sample, resulting in a total sample of 133 participants.

Participants were self-identified trans adults living in Canada who ranged in age from 18 to 75 years (*M* = 36.75, *SD* = 13.01). A wide variety of identities were reported by participants, with the majority of participants identifying as transgender (51.1 %, *n* = 68), trans (50.4 %, *n* = 67), transexual/transsexual (45.1 %, *n* = 60), man or boy (37.6 %, *n* = 50), and woman or girl (37.6 %, *n* = 50) (see Table 1). The categories were not presented in a mutually-exclusive manner. In a question separate from identity, an almost equal number of participants reported being on the FTM spectrum (42.1 %, *n* = 56) and on the MTF spectrum (44.4 %, *n* = 59), while 2.3 % reported having an intersex condition (*n* = 3) and 11.3 % offered answers under *other* (*n* = 15). Although the recruitment material stated that trans adults were invited to participate in the study, it is important to note that many participants underlined the fact that they were people of trans experience, with trans experience, or who have transitioned, without necessarily identifying as trans.

**Table 1:** Demographic information

	*n*	%
Current identity		
Man or boy	50	37.6
Woman or girl	50	37.6
Trans	67	50.4
Transgender	68	51.1
Transexual/transsexual	60	45.1
FTM	36	27.1
MTF	39	29.3
Someone on the FTM spectrum	20	15.0
Someone on the MTF spectrum	23	17.3
Genderqueer	33	24.8
Two-spirit	10	7.5
Transman	33	24.8
Transwoman	41	30.8
Man of trans experience	11	8.3
Woman of trans experience	10	7.5
Androgyne	11	8.3
Woman; boy; gender blender; bi-gender; polygender/pangender; cross-dresser; transvestite; intersexual; drag king^a^	40	30.4
Other: e.g., Ft other; gender bent; third gender; gender fucker; trans woman	14	10.6
Current sexual orientation		
Lesbian	32	24.1
Gay	14	10.5
Bisexual	35	26.3
Queer	54	40.6
Dyke	13	9.8
Fag/faggot	15	11.3
Heterosexual/straight	31	23.3
Pansexual	20	15.0
Two-spirit; same-gender loving; asexual^a^	21	15.8
Not sure or questioning	16	12.0
Other: e.g., heteroflexible	12	9.0
Ethnocultural background		
White/Caucasian	45	34.9
European	21	16.3
Canadian or French-Canadian or Québécois(e)	19	14.7
European-Canadian	15	11.6
Jewish	6	4.7
Asian	6	4.7
Bi/multi-ethnicity	6	4.7
Aboriginal descent; Latino/Latina; Middle-Eastern; other^a^	11	8.6
Religious identity		
Buddhist	9	6.8
Catholic	16	12.0
Jewish	9	6.8
Protestant	9	6.8
Aboriginal spirituality; Hindu; Muslim; Wiccan^a^	12	9.1
Agnostic or atheist	61	45.9
Other	44	33.1
Highest level of education to date		
Less than high school; high school or equivalent; trade school; other^a^	30	22.6
Some college	29	21.8
College	23	17.3
Undergraduate degree	27	20.3
Master’s degree	18	13.5
Doctoral degree	6	4.5
Current employment situation		
Work full-time	45	33.8
Work part-time	19	14.3
Self-employed	23	17.3
Unemployed	23	17.3
Unable to work	14	10.5
Student	37	27.8
Retired; homemaker/stay at home parent^a^	8	6.1
Other	15	11.3
Personal yearly income		
Below $10,000	36	27.1
Between $10,001 and $20,000	29	21.8
Between $20,001 and $30,000	14	10.5
Between $30,001 and $40,000	10	7.5
Between $40,001 and $50,000	10	7.5
Between $50,001 and $60,000	7	5.3
Between $60,001 and $70,000	10	7.5
Over $70,000	14	10.5
Relationship status		
Single (never married)	43	32.3
In one or more relationship(s)/dating, living apart	31	23.3
In one or more relationship(s)/dating, living together	11	8.3
Married	19	14.3
Common law union	12	9.0
Separated; divorced^a^	15	11.3
Other	2	1.5

Categories in current identity, current sexual orientation, ethnocultural background, and current employment situation were not mutually exclusive; thus, sums may be greater than 100 %

^a^Categories combined due to low cell size

The majority of participants reported living in Québec (33.8 %, *n* = 45), 32.3 % reported living in Ontario (*n* = 43), 23.3 % in British Columbia (*n* = 31), and 9.0 % (*n* = 12) elsewhere in Canada (Alberta, Saskatchewan, Manitoba, and New Brunswick). Most participants completed the study in English (82.7 %, *n* = 110), with the rest participating in French. Participants reported a variety of sexual orientations (for details, see Table 1). Participants were asked to identify their ethnocultural background with no predetermined answers offered; as such, responses were read through and grouped according to common race, ethnicity, culture, or geographic location. The majority of participants identified their ethnocultural background as White/Caucasian (see Table 1). Participants reported living in different areas: 75.2 % of participants reported living in an urban area (*n* = 100), 17.3 % reported living in a suburban area (*n* = 23), and 6 % reported living in a rural area. Numerous participants reported being agnostic or atheist (45.9 %, *n* = 61) while other participants reported a variety of religious identities (see Table 1). The majority of participants did not consider themselves to be practicing members of their current religious group (46.6 %, *n* = 62) while 22.6 % did (*n* = 30), and the remaining 30.8 % (*n* = 41) either did not answer the question or answered under *other* (e.g., “Sometimes,” “It’s complicated”). Over 90 % of participants reported being Canadian citizens (91.7 %, *n* = 122). A total of 37 % of participants (*n* = 49) reported living with either a visible or invisible disability or disabilities and/or a chronic illness. The majority of participants reported having completed some college (21.8 %, *n* = 29), having completed college (17.3 %, *n* = 23), or having obtained an undergraduate degree (20.3 %, *n* = 27) (see Table 1). Most participants reported working full-time (33.8 %, *n* = 45) (see Table 1), and the majority of participants reported earning less than $10,000 per year in personal yearly income (27.1 %, *n* = 36), with 19 of these participants also being students (see Table 1). Lastly, almost one third of participants reported being single/never married (32.3 %, *n* = 43) (see Table 1).

### Measures

Measures that were not available in a standardized French version were translated into French and subsequently validated by back-translation. A company specializing in English–French and French–English translation did both of these translations.

#### Demographic Information

A demographic form asked participants to self-identify various demographic characteristics such as age, gender identity, sexual orientation, ethnocultural background, and relationship status. As *trans* is an umbrella term that is used to describe many different kinds of people, participants were asked to indicate how they currently identify using a list of 26 possible identities, with the additional choice of *other*. Choices were not mutually exclusive.

#### Optimism

Optimism was assessed with Scheier, Carver, and Bridges’ (1994) Life Orientation Test Revised (LOT-R). The LOT-R is a 10-item self-report scale that measures generalized optimism. The measured optimism was not related to any event(s) in particular; participants were simply asked to report the frequency of optimism they felt in general. Participants rated items such as “In uncertain times, I usually expect the best” and “If something can go wrong for me, it will” (reverse coded) on a 5-point Likert-type scale (1 = *I disagree a lot* to 4 = *I agree a lot*). Four items were fillers and therefore not coded. Three items were reverse coded and participants’ responses were summed, with higher scores indicating higher levels of optimism. Cronbach’s alpha for the original scale was .78 and, in the current study, it was .85. Validity was evidenced by both exploratory and confirmatory factors analyses in which all six items loaded onto one factor (with results from the confirmatory analysis supporting both a one and two factor model). Furthermore, scores on the LOT-R were correlated in the expected directions with scores on conceptually-related scales (Scheier et al., 1994).

#### Social Support

Perceived social support was assessed with Procidano and Heller’s (1983) Perceived Social Support Scale from Friends and Family (PSS-Fr and PSS-Fa). The PSS-Fr and PSS-Fa are two separate self-report questionnaires, consisting of 20 items each that assess the extent to which participants perceive their family and friends meet their needs for support, feedback, and information. Participants answered *Yes*, *No*, or *Don’t know* to items such as “I rely on my friends for emotional support” (in the PSS-Fr) and “There is a member of my family I could go to if I were just feeling down, without feeling funny about it later” (in the PSS-Fa). Items were scored appropriately (*Yes* = 1, and *No* = 0, *Don’t know* = 0) and six items in the PSS-Fr and five items in the PSS-Fa were appropriately reverse coded (*Yes* = 0, and *No* = 1, *Don’t know* = 0). The ratings were summed for each scale, with higher scores indicating greater levels of perceived social support. Cronbach’s alpha was .88 for the PSS-Fr scale and .90 for the PSS-Fa scale. In the current study, Cronbach’s alpha was .89 for the PSS-Fr scale and .94 for the PSS-Fa scale. Validity was evidenced in both the PSS-Fr and PSS-Fa by scores on these measures being significantly correlated in the expected directions with scores on measures of distress, psychopathology, and social network availability (Procidano & Heller, 1983).

#### Suicide Resilience

Suicide resilience was assessed with Osman et al.’s (2004) Suicide Resilience Inventory 25 (SRI-25). The SRI-25 is a 25-item self-report measure used to assess factors that help defend against suicidal thoughts and behaviors. It is comprised of three subscales, two of which (External Protective and Emotional Stability) include the assessment of suicide-specific resilience. The External Protective subscale assesses people’s positive perceptions or beliefs that they are able to seek help from those close to them should they experience suicidal thoughts; the Emotional Stability subscale assesses people’s positive perceptions or beliefs that they are able to resist acting on suicidal thoughts when experiencing them. The third subscale, the Internal Protective subscale, assesses people’s satisfaction with life and positive feelings about themselves overall. Participants rated items such as “I like myself” (Internal Protective Factors subscale), “I can deal with the emotional pain of rejection without thinking of killing myself” (Emotional Stability Factors subscale), and “I could openly discuss thoughts of killing myself with people who are close to me, when it is necessary” (External Protective Factors subscale). The ratings were averaged by subscale, with higher total scores indicating greater resilience against committing suicide. Cronbach’s alphas were above .90 for the total scale and each subscale in both the original and current studies.

Validity was evidenced by confirmatory factors analyses in which all items significantly loaded onto one of three factors, with the exception of one item that loaded onto its primary factor and one other factor (Osman et al., 2004). Furthermore, the SRI-25 successfully differentiated participants with past suicidal ideation or behavior from participants with no reported history of suicidal ideation and behavior as measured by the Suicidal Behaviors Questionnaire Revised (Osman et al., 2001), a validated measure of suicidal ideation and attempts (Osman et al., 2004). Suicide resilience also was shown to negatively correlate with suicidal ideation in a diverse sample of college students (Rutter et al., 2008).

#### Reasons for Living

Reasons for living were assessed with Linehan et al.’s (1983) Reasons for Living Inventory (RFL). The RFL is a 48-item self-report scale that measures participants’ endorsement of certain reasons for living. It is used to measure different reasons for living in both participants who have experienced suicidal ideation and those who have not. The RFL is based on the premise that adaptive beliefs and expectations can serve as factors that protect individuals against suicidal ideation and behavior. The RFL is comprised of six subscales that each measure different types of reasons for living: survival and coping beliefs (beliefs about the value of life and one’s coping capabilities), responsibility to family (beliefs about one’s responsibility to one’s family as a reason for staying alive), child-related concerns (reasons for staying alive related to one’s child or children), fear of suicide (beliefs about suicide relating to one’s apprehension and fear of committing the act), fear of social disapproval (fear of what other people’s perception would be if one were to commit suicide), and moral objections (religious and moral beliefs related to suicide).

Participants rated items such as “I believe I can find other solutions to my problems” (Survival Coping Subscale) and “The effects on my children could be harmful” (Child-Related Concerns Subscale) on a 6-point Likert-type scale (1 = *Not at all important as a reason for not killing myself* to 6 = *Extremely important as a reason for not killing myself*). The ratings were averaged by subscale, with higher scores indicating greater endorsement of that category of reasons for living. Cronbach’s alpha in the original scale was not reported by Linehan et al. (1983); however, Osman et al. (1993) showed it to be .89 for the total scale, and ranging from .79 to .92 for the subscales. In the current study, Cronbach’s alpha was .93 for the total scale; .95 for the Survival Coping Beliefs subscale, .91 for the Responsibility to Family subscale; .88 for the Child-Related Concerns subscale; .84 for the Fear of Suicide subscale; .81 for the Fear of Social Disapproval subscale; and .75 for the Moral Objection subscale. Validity was evidenced by four exploratory factors analyses; six factors emerged in all four analyses and items with ambiguous factor loadings were subsequently dropped. Furthermore, reasons for living have been assessed in a wide array of samples and have been found to differentiate between past suicide attempters and non-attempters in the general population (Linehan et al., 1983), as well as in samples of individuals hospitalized for mental health reasons (Linehan et al., 1983; Lizardi et al., 2007; Malone et al., 2000).

#### Suicidal Behavior

Suicidal ideation and behavior was assessed with Osman et al.’s (2001) Suicidal Behaviors Questionnaire Revised (SBQ-R), which is an adaptation of Linehan’s (1981) longer SBQ. The SBQ-R is a four-item self-report scale that measures distinct aspects of suicidal behavior. It measures lifetime suicidal ideation and/or behavior (“Have you ever thought about or attempted to kill yourself?”), the frequency of suicidal ideation in the past year (“How often have you thought about killing yourself in the past year?”), the communication of suicidal thoughts to others (“Have you ever told someone that you were going to commit suicide, or that you might do it?”), and the likelihood of attempting suicide in the future (“How likely is it that you will attempt suicide in the future?”). Participants rated these items along their corresponding response scales and the ratings were summed for a total scale score. Higher scores indicate higher levels of risk for suicidal behavior. Summing participants’ responses for a total scale score is one of two ways (the other is to use a single item) the SBQ-R has been validated (Osman et al., 2001) and is consistent with existing literature (e.g., Bryan, Cukrowicz, West, & Morrow, 2010; Charbrol, Chauchard, & Girabet, 2008; Johnson, Gooding, Wood, & Tarrier, 2010; Osman et al., 2002; Taylor, Wood, Gooding, & Tarrier, 2010). Cronbach’s alpha was .76 in both the original and current studies. Validity was evidenced by total SBQ-R scores accurately differentiating between suicidal and non-suicidal subgroups in separate samples of clinical and nonclinical adolescents and adults (Osman et al., 2001). In the clinical samples, the SBQ-R accurately differentiated between individuals who were hospitalized due to a serious threat of suicide or a suicide attempt and reported recent ideation/attempt (suicidal) and those who were hospitalized due to another mental health problem and reported no recent ideation/attempt (non-suicidal). In the non-clinical samples, the SBQ-R accurately differentiated between individuals who reported recent ideation/attempt (suicidal) and those who reported no recent ideation/attempt (non-suicidal).

### Procedure

Recruitment e-mails were sent to participants via LGBT and trans LISTSERVs and organizations. Data collection was conducted online using a secure survey platform and participation was anonymous. To assure complete anonymity, the survey platform was configured to not save IP addresses or date/time stamp of completed entries. Participants had the option of participating in English or in French. Participants were invited to register their informed consent and complete a series of questionnaires. Participants had the choice to withdraw their consent at any point while completing the series of questionnaires. They could do so in one of two ways: they could stop the survey at any point by clicking on the “Exit and clear survey” icon or, once at the end of the survey, they could click on “Exit and clear survey” rather than “Submit.” Both of these actions ensured that their answers were not included in the study. A paper-and-pencil version of the questionnaires was available upon request, with a postage-paid return envelope included. To encourage participation in the study, participants could opt to be included in a lottery for three prizes of $100 each. If participants opted into the draw, they were asked to enter their e-mail address for notification in a separate online survey in order to protect the anonymity of their previous responses. The study received approval by the Research Ethics Board of McGill University.

An anonymous, online data collection methodology was used for two reasons. Online recruitment and participation in research has been described in the literature as a suitable and appropriate method of reaching participants belonging to hidden populations, including trans populations (Miner, Bockting, Swinburne Romine, & Raman, 2012; Rhodes, Bowie, & Hergenrather, 2003). Furthermore, as the current research project collected data nationally, in-person data collection was impossible. As participation was anonymous and IP addresses were not collected, the possibility exists that participants completed the questionnaires twice, producing duplicate submissions. The chance of this occurring was low, however, as participation took 45–60 min and participants could enter a lottery rather than receive direct compensation. Data were nonetheless verified manually and no two participants entered the same demographic information. Furthermore, all participants met the eligibility criteria; all participants were required to enter their age and all participants indicated their identity under the broader term of *trans*.

### Data Analysis

Mean item substitution was used when a minimum of 80 % of the items on a given subscale was answered, with two exceptions. Mean item substitution was not used for missing data on the SBQ-R due to the scale being comprised of four items that measured four distinct aspects of suicidal behavior. Furthermore, mean item substitution was not used for missing data on the RFL-48, as per the specific scoring guidelines for that scale. Mahalanobis distance was used to detect multivariate outliers; the data set contained no such outliers. All analyses were carried out using SPSS 17. Values of variation inflation factors (VIF) and tolerance were examined in order to detect multicollinearity. The generally accepted rule of a VIF value above 10 and/or a tolerance value below .2 indicating multicollinearity was applied; no multicollinearity was detected.

Data distributions were evaluated prior to conducting the analysis; certain data were either positively or negatively skewed. As recommended by Tabachnick and Fidell (2007), data that were moderately skewed were transformed using a square root transformation; data that were substantially skewed were transformed using a logarithm transformation; and data that were severely skewed were transformed using an inverse transformation. Data that were negatively skewed were reflected in addition to being transformed; reflection refers to the process of converting negatively skewed data to positively skewed data for the purpose of transformation. Specifically, data from the SRI-25 External Protective subscale were transformed using an inverse transformation; data from the RFL Fear of Suicide subscale were transformed using a logarithm transformation; and data from the Perceived Social Support from Friends Scale were reflected in addition to being transformed using a square root transformation.

## Results

### Suicidal Behavior Scores

All but one participant (*n* = 132) answered all four questions on the SBQ-R. The mean total score was 9.8 (*SD* = 3.7) out of a possible range of 3–18. Although not used in the context of this research, the SBQ-R has two validated cutoff scores indicating risk for suicidal behavior: scores ≥7, validated with undergraduate students and scores ≥8, validated with adult inpatients. Score distributions of the SBQ-R are shown in Table 2. Preliminary analyses (i.e., *t*-tests and ANOVA) examined whether participants’ suicidal behavior scores differed significantly according to demographic variables. Results indicated that suicidal behavior scores did not differ significantly as a function of demographic variables.^2^

**Table 2:** Suicidal ideation and behavior

	*n*	%
Lifetime suicide ideation/attempt		
Never	11	8.3
It was just a brief passing thought	28	21.1
I have had a plan at least once to kill myself	59	44.4
I have attempted to kill myself	35	26.3
Frequency of suicide ideation in past year		
Never	34	25.6
Rarely (1 time)	23	17.3
Sometimes (2 times)	23	17.3
Often (3–4 times)	21	15.8
Very often (5 or more times)	32	24.1
Communication of suicidal thoughts to others		
No	61	45.9
Yes, at one time, but did not really want to die	26	19.5
Yes, at one time, and really wanted to die	15	11.3
Yes, more than once, but did not want to do it	10	7.5
Yes, more than once, and really wanted to do it	20	15.0
Likelihood of suicidal behavior in the future		
Never	20	15.0
No chance at all	19	14.3
Rather unlikely	48	36.1
Unlikely	22	16.5
Likely	15	11.3
Rather likely	6	4.5
Very likely	3	2.3

If a participant answered the fourth question with “likely,” “rather likely” or “very likely,” the following message appeared: “Although the measures you filled out are not diagnostic tools, based on this information, you are highly encouraged to call the National Suicide Prevention Lifeline at 1-800-273-8255 and to consult a mental health professional. Always consult with a trained suicide prevention volunteer and/or mental health professional if you are experiencing serious thoughts of ending your life. Furthermore, if you are currently experiencing serious thoughts of ending your life, you should immediately go to the emergency department of your local hospital to seek help”

### Correlations Between Suicidal Behavior and Predictor Variables

The means, SD, and correlations between variables are shown in Table 3. Optimism and perceived social support from family were significantly negatively correlated with suicidal behavior. Perceived social support from friends was significantly positively correlated with suicidal behavior; however, the data from this variable were transformed and reflected prior to the analysis. The direction of the correlation should, therefore, be reversed when looking at the results. All three suicide resilience factors (internal protective factors, emotional stability, and external protective factors) were also significantly negatively correlated with suicidal behavior, as were certain reasons for living (survival coping beliefs, responsibility to family, and child-related concerns).

**Table 3:** Correlations between suicidal behavior, age, optimism, perceived social support, suicide resilience, and reasons for living

Variable	1	1a	1b	1c	1d	2	3	4	5	6
1. SBQ-R total score	–									
1.a. Lifetime suicide ideation/attempt	.71***	–								
1.b. Ideation in past year	.85***	.42**	–							
1.c. Communication of suicidal thoughts to others	.66***	.42***	.44***	–						
1.d. Likelihood of suicidal behavior in the future	.85***	.53***	.60***	.40***	–					
2. Age	−.01	.08	−.09	−.08	.06	–				
3. Optimism	−.53***	−.41***	−.46***	−.28**	−.45***	.08	–			
4. Perceived support from friends^a^	.17*	.14	.17*	.02	.16*	.11	−.30***	–		
5. Perceived support from family	−.42***	−.28**	−.41***	−.23**	−.35***	.06	.43***	−.23**	–	
6. Internal protective	−.55***	−.34***	−.47***	−.36***	−.50***	.09	.74***	−.41***	.39***	–
7. Emotional stability	−.64***	−.38***	−.50***	−.35***	−.67***	.04	.59***	−.34***	.38***	.69***
8. External protective^b^	−.45***	−.29***	−.34***	−.27**	−.46***	−.04	.54***	−.44***	.36***	.53***
9. Survival coping beliefs	−.49***	−.31***	−.37***	−.21**	−.55***	.00	.43***	−.24**	.25**	.54***
10. Responsibility to family	−.17*	−.09	−.20*	.06	−.21*	.19*	.19*	−.10	.49***	.14
11. Child-related concerns	−.24**	−.26**	−.15*	−.11	−.24**	.30***	.14	.04	.08	.13
12. Fear of suicide^b^	.07	.08	.00	.10	.07	−.15*	−.17*	.08	.02	−.14
13. Fear of social disapproval	−.04	−.01	−.01	.09	−.14	−.15*	−.10	.09	.08	−.05
14. Moral objections	−.10	−.02	−.07	−.03	−.15	.00	.02	.02	.11	.07
Mean	9.76	2.89	2.94	1.76	2.17	36.75	12.83	12.38^c^	7.83	4.29
SD	3.74	.91	1.55	.80	1.46	13.01	6.14	5.45^c^	6.58	1.15

Variable	7	8	9	10	11	12	13	14
7. Emotional stability	–							
8. External protective^b^	.64***	–						
9. Survival coping beliefs	.54***	.39***	–					
10. Responsibility to family	.17*	.21**	.25**	–				
11. Child-related concerns	.12	.09	.32***	.38***	–			
12. Fear of suicide^b^	−.15*	−.11	.04	.09	−.01	–		
13. Fear of social disapproval	−.10	−.04	.16*	.40***	.17*	.30***	–	
14. Moral objections	.06	−.07	.29***	.20*	.16*	.25**	.34***	–
Mean	4.66	4.70^c^	4.01	3.56	2.64	2.61^c^	2.37	1.56
SD	1.16	1.23^c^	1.16	1.51	1.87	1.22^c^	1.37	.99

*n* = 127

* *p* < .05, ** *p* < .01, *** *p* < .001

^a^Transformed distribution with a reflection. Interpretation of the direction of the results should therefore be reversed

^b^Transformed distribution

^c^Means and standard deviations from non-transformed distribution for comparison purposes

### Regression Model

As mentioned above, mean item substitution was used when a minimum of 80 % of the items on a given subscale was answered. Six cases had less than 80 % of items answered per subscale and were therefore deleted listwise, resulting in a final sample of 127 participants included in the regression model. Hierarchical multiple regression analysis was used to predict suicidal behavior from protective factors. Specifically, a three-block model was tested to determine whether protective factors would negatively predict variance in participants’ suicidal behavior (i.e., total SBQ-R score, comprised of lifetime suicidal ideation and/or behavior, suicidal ideation in the past year, threats of suicide attempt, and likelihood of suicidal behavior in the future). As previously mentioned, being younger than 25 years old has been found to be an independent predictor of suicide attempts in trans individuals (Clements-Nolle et al., 2006). Age was entered in the first block in order to control for the effects of this variable. Due to no significant difference being found in suicidal behavior scores across participants who identified as someone on the FTM spectrum, on the MTF spectrum, or as having an intersex condition/*other*, this variable was not included in the regression model.

Blocks 2 and 3 were decided upon using Cohen’s (1986) guidelines regarding hierarchical multiple regression. Cohen stipulates that “the order of precedence is such that one is prepared to assume that no factor coming later in the series can causally effect one coming earlier” (p. 39). In theory, optimism, social support from friends, and social support from family could causally effect reasons for living and suicide resilience. Therefore, the former three variables were entered simultaneously in the second block and the latter variables were entered simultaneously in the third block.

Once the variance in suicidal behavior due to age was controlled for, optimism, social support from friends, and social support from family significantly negatively predicted variance in participants’ suicidal behavior, Δ*R* ^2^ = .33, change in *F*(3, 122) = 19.72, *p* < .001. Once the variance in suicidal behavior due to age, optimism, social support from friends, and social support from family was controlled for, suicide resilience and reasons for living contributed significant variance to participants’ suicidal behavior, Δ*R* ^2^ = .19, change in *F*(9, 113) = 5.17, *p* < .001.

Results of the full hierarchical regression model are shown in Table 4. Of the variables included in the second block, perceived social support from family significantly and negatively predicted participants’ suicidal behavior, *t* = −2.72, *p* < .01. Of the variables included in the third block, one suicide resilience factor (emotional stability, *t* = −3.80, *p* < .001) and one RFL (child-related concerns, *t* = −2.11, *p* < .05) significantly and negatively predicted participants’ suicidal behavior.

**Table 4:** Hierarchical multiple regression analysis predicting suicidal behavior from optimism, perceived social support, suicide resilience, and reasons for living

Predictor	*B*	*SE B*	B	*t*	*R* ^2^	Adjusted *R* ^2^	*R* ^2^ change	*F* change	*df*
Step 1					.00	−.01	.00	.01	1, 125
Age	.02	.02	.06	.85					
Step 2					.33	.31	.33	19.72***	3, 122
Optimism	−.07	.06	−.12	−1.16					
Perceived support from friends^a^	−.39	.29	−.11	−1.38					
Perceived support from family	−.13	.05	−.23	−2.72**					
Step 3					.52	.47	.19	5.17***	9, 113
Internal protective	−.26	.38	−.08	−.67					
Emotional stability	−1.29	.34	−.40	−3.80***					
External protective^b^	−.29	1.35	−.02	−.22					
Survival coping beliefs	−.37	.29	−.12	−1.30					
Responsibility to family	.34	.23	.14	1.52					
Child-related concerns	−.32	.15	−.16	−2.11*					
Fear of suicide^b^	.09	1.23	.01	.07					
Fear of social disapproval	−.21	.22	−.08	−.96					
Moral objections	.07	.28	.02	.26					

*n* = 127. Regression coefficients reported from final step

* *p* < .05, ** *p* < .01, *** *p* < .001

^a^Transformed distribution with a reflection. Interpretation of the direction of the results should therefore be reversed

^b^Transformed distribution

As social support from friends was not found to be a significant predictor of suicidal behavior, a secondary analysis was conducted in which scores for social support from friends and social support from family were compared. Participants reported significantly less social support from family than from friends, *t*(132) = 7.36, *p* < .001.

## Discussion

The present study was the first to examine suicide protective factors among trans adults. Given the high rates of suicide attempts in trans communities, identifying factors that protect trans individuals against suicidal ideation and behavior appears timely. Results indicated that perceived social support from family, emotional stability (an aspect of suicide resilience), and child-related concerns (a reason for living) were associated with lower suicidal behavior scores in trans individuals. Taken together, these results provided support for the hypothesis that there were significant relationships between some factors typically found to protect cis individuals from suicidal behavior and trans individuals’ suicidal behavior.

The suicide resilience factor of emotional stability was found to be an important protective factor against suicidal behavior. It should be noted that the mean scores for all three suicide resilience subscales were lower than would be expected in a convenience sample. The subscale means for participants in the current study were 4.3 (*SD* = 1.2), 4.7 (*SD* = 1.2), and 4.7 (*SD* = 1.2) for the Internal Protective subscale, the Emotional Stability subscale, and the External Protective subscale, respectively. These means were lower than the means found in both Osman et al. (2004) and Rutter et al. (2008). The finding that the current sample had lower than average rates of suicide resilience may have meaningful implications for practitioners working with clients who are trans or who have a trans history. The delivery of interventions that are aimed at increasing suicide resilience may prove to be beneficial with certain clients, particularly those experiencing suicidal ideation.

In addition to emotional stability being an important protective factor against suicidal behavior, child-related concerns emerged as an important protective factor. This finding was consistent with studies that include both a general population sample and a sample of hospitalized individuals. Indeed, Linehan et al. (1983) found that child-related concerns differentiated between individuals who had no reported history of suicidal behavior or brief ideation and those who had a reported history of serious ideation or behavior in a general population sample. They also found that child-related concerns differentiated between individuals who were currently non-suicidal and those who were currently suicidal in a sample of hospitalized individuals. Lastly, child-related concerns also differentiated between those who were experiencing current ideation and those who had attempted suicide in the same group of hospitalized individuals.

Perceived social support from family significantly and negatively predicted participants’ suicidal behavior scores. This finding was consistent with the extant research regarding social support and suicide ideation and/or attempts. Marion and Range (2003) found that perceived social support significantly and negatively predicted suicide ideation in a sample of African American college students. In a more recent study, Chioqueta and Stiles (2007) found that social support significantly and negatively predicted suicidal ideation, above and beyond hopelessness and depression, in a sample of 314 university students. Social support, as measured by specific questions regarding distinct areas of support, was also associated with both lower rates of suicidal ideation and attempts in a sample of 2,255 youth who had had at least one same-sex sexual experience (Eisenberg & Resnick, 2006). Of particular interest, social support from family, as measured by a group of questions assessing family connectedness, was shown to be an important protective factor among these youth. Both male and female youth who reported high levels of family connectedness had almost half the odds of suicidal ideation and attempts than youth who reported lower levels of family connectedness, with the exception of males who had .60 the odds of suicidal attempts. Likewise, Bouris et al. (2010) conducted a systematic review of the literature regarding parental influences on the health of LGB youth and found that parental closeness and support was a suicide protective factor among these youth.

Participants reported significantly less social support from family than from friends. This finding was in line with current literature that underlines the occurrence of rejection of trans individuals from their families of origin due to transphobia and transprejudice (Association of Lesbian, Gay, Bisexual, and Transgender Issues in Counseling, 2009). Despite the fact that trans participants in the current sample perceived that they received much less social support from their families than from their friends, it was the social support from their families that was significantly associated with lower rates of suicidal behavior. Given that there were high scores and a restricted range seen in the Perceived Social Support from Friends Scale responses, the non-significant associations between perceived social support from friends and suicidal behavior may be due to a ceiling effect. However, given that perceived social support from families appears to be a suicide protective factor, the development of interventions aimed at the families of trans individuals’ may prove to have beneficial outcomes for the trans individuals themselves.

That having been said, it must be stressed that the data in question were cross-sectional and thus causal relationships between variables cannot be assumed. Alternate explanations regarding the association between perceived social support from family and suicidal behavior are entirely feasible. For example, the association between the two variables may operate in the opposite direction; individuals with lower suicidal behavior scores may have the perception that they receive higher levels of social support from family. There is also the possibility one or more other variables moderate the relationship between perceived social support and suicidal behavior.

### Limitations

As mentioned above, data were cross-sectional and obtained through questionnaires; causal relationships between variables therefore cannot be assumed. Furthermore, as there was no comparison group in the current study, interpretation of the lower scores on some of the measures (e.g., SRI-25) is tentative at best. Moreover, results may not be generalizable due to the small sample size based on convenience, non-random sampling, as well as the somewhat homogeneous ethnocultural backgrounds of the participants. In addition, although a paper version of the survey was available to those who requested it, all recruitment was conducted online. Therefore, individuals who did not have access to a computer, the internet, or who were not on the LGB and trans LISTSERVs on which the recruitment emails were distributed most probably did not see the recruitment advertising and were not able to participate. Lastly, it was the first time that the measures were administered to trans individuals. Though many of the correlations found among cis individuals were also found in the current study, the validity of the measures with trans populations should continue to be explored. In addition, only suicide resilience, reasons for living, and selected general suicide protective factors were assessed. Therefore, one should be cautious against considering only these protective factors; factors associated with lower suicidal behavior scores in the current study are but some factors out of a plethora of potential protective factors already identified for cis individuals. Lastly, the current study was conceptually limited due to the fact that risk factors were not assessed, which made the mediating or buffering effect of the protective factors impossible to analyze.

Despite these limitations, we suggest that the current study was a first step in the identification of suicide protective factors among trans individuals. Although it was not possible to examine the mediating or buffering role of the protective factors against risk factors, the analysis of protective factors through negative and significant predictions of variance in suicidal behavior scores was in line with common practices for identifying suicide protective factors (e.g., Marion & Range, 2003). It is our hope that much more research will be conducted on this topic and that the results will be used to inform the practices of mental health workers, medical doctors, and suicide prevention workers working with trans clients.

### Directions for Future Research

Future research should use conceptual models that take risk factors, in addition to protective factors, into account. Such can be done in a number of ways, such as testing integrative models (Rutter, 2008) or by investigating the moderating effects of protective factors on the relationship between risk factors and suicidal behavior. Furthermore, in order to avoid the limitations associated with cross-sectional designs, future research would benefit from longitudinal designs. However, it should be noted that although this recommendation is simple to make in theory, it may be rather inappropriate to carry out in practice due to the ethical and legal concerns that must be considered when conducting research regarding suicidal ideation and/or attempts.

Future research may also benefit from examining the relationships between protective factors and suicidal ideation and/or attempts in different groups of trans individuals in order to understand potential important within-group differences. For example, although no significant differences were found in suicidal behavior scores across participants who reported being on the FTM spectrum, the MTF spectrum, or being a person with an intersex condition/*other* in the current sample, this may not be the case in other samples and thus the investigation of differences among protective factors across groups of trans individuals may be warranted. Different factors may also be protective for trans people of color than for White trans individuals, and for LGB trans individuals than for heterosexual trans individuals, and for young adult trans individuals than for older adult trans individuals. Lastly, future research would also benefit from the exploration of protective factors that were not included in the present study and/or factors that may be protective specifically for trans individuals. As the investigation of protective factors is in its infancy, the above future directions will allow for the development of specific and appropriate suicide prevention models and interventions for suicidal trans individuals.

## Footnotes

1 In context of the current article, trans is used as an umbrella term that refers to a wide variety of self-identities, including, but not limited to, trans, transgender, transexual/transsexual, genderqueer, Two-Spirit, and people with trans histories.

2 Information regarding these analyses and results is available from the corresponding author upon request.
